# Case Report: Unusual Cause of Chest Pain: A Multi-Image Assessment of a Cardiac Mass

**DOI:** 10.3389/fcvm.2022.889406

**Published:** 2022-06-23

**Authors:** Javier Serrano-Roman, Santiago Saenz-Ancira, Jose C. Armendariz-Ferrari, Valente Fernandez-Badillo, Enrique Solorzano-Pinot, Adrian Espejel-Guzman, Joaquin Berarducci, Alberto Aranda-Fraustro, Mauricio Garcia-Cardenas, Nilda Espinola-Zavaleta

**Affiliations:** ^1^Department of Nuclear Cardiology, National Institute of Cardiology Ignacio Chavez, Mexico City, Mexico; ^2^Department of Clinical Cardiology and Echocardiography, Hospital Nacional Hipolito Unanue, Lima, Peru; ^3^Department of Pathology, National Institute of Cardiology Ignacio Chavez, Mexico City, Mexico; ^4^Department of Echocardiography, ABC Medical Center I.A.P, Mexico City, Mexico

**Keywords:** myxoma, tumor, neovascularization, echocardiography, computed tomography angiography

## Abstract

Myxomas represent the most common benign primary cardiac tumor, they usually grow out of the interatrial septum into the left atrium with a pedunculated base. Intracardiac masses can be found incidentally on imaging studies, but symptomatology may arise secondary to the mass effect, embolization, and valvular function impairment. We present the case of a 75-year-old woman who arrived at the emergency department with atrial fibrillation and NSTEMI segment elevation myocardial infarction (NSTEMI) secondary to a highly vascularized neoplasm visible by coronary angiography and angiotomography. Scarce reports show high quality multi-imaging assessment of significantly vascularized myxomas with such atypical presentation. High-definition imaging studies played a fundamental role in the surgeon’s management of a mass with a complex neovascularization.

## Introduction

Primary cardiac tumors are a rare entity with benign etiology in approximately 90%. Myxomas represent the most frequent type with almost 80% and tend to be located in the left atrium (1, 2). The clinical presentation depends on the location and size of the tumor, and whether it is associated with valvular insufficiency, obstruction, or embolic events. Such signs and symptoms may differ among them, such as dyspnea, pulmonary edema, auscultatory findings, and in cases of right-sided masses, foot edema, hepatomegaly, and even ascites.

Case series have shown a relatively high rate of neovascularization in left atrial myxomas (LAM), with an increased use of non-invasive imaging modalities such as Coronary Computed Tomography Angiography (CCTA) in the preoperative evaluation and characterization of highly vascularized neoplasms (3–5). We present a case in which a multi-imagen assessment was performed in order to understand the tumor’s enormous blood supply, which influenced the surgical decisions and resulted in the complete recovery of the patient.

## Case Description

A 75-year-old female was admitted into the emergency department due to acute chest pain and dyspnea. There was no history of cardiovascular risk factors, prior heart disease, or trauma and the patient was otherwise in good health. On physical examination, jugular ingurgitation and bilateral rales in pulmonary fields were identified, heart sounds were arrhythmic and tachycardic (150 bpm) without murmurs. Other vital signs showed hypotension (80/60 mmHg) and tachypnea (26 rpm). Chest x-ray depicted cardiomegaly, left and right atrial enlargement and signs of pulmonary congestion. An initial electrocardiogram demonstrated atrial fibrillation (AF) and ST segment depression in the anterolateral wall (V1-V5), suggestive of a non-ST segment elevation myocardial infarction (NSTEMI) (Table 1). Baseline cardiac enzymes assessment surpassed the upper limit for CK 2919 U/L, CK-MB 57.5 U/L, and troponin I 0.058 ng/mL.

**TABLE 1:** Timeline.

**Day 0** ● A 75-year-old female was admitted into the emergency department due to acute chest pain and dyspnea. ● *Chest x-ray* evidenced cardiomegaly, atrial enlargement and pulmonary congestion. ● *Electrocardiogram*. Atrial fibrillation, non-ST segment elevation myocardial infarction. ● *Laboratory*. CK, CK-MB, and troponin I with increased values. ● *Urgent coronary angiography* showed abnormal neovascularity inside the left atrium and coronary artery disease. **Day 1** ● *Transthoracic and transesophageal echocardiogram* evidenced an enormous and highly vascularized mass in the left atrium. **Day 2** ● *Coronary computed tomography angiography* revealed the complex network of blood vessels inside the mass, which arised from the right coronary and circumflex arteries. **Day 4** ● Artery ligation and tumor resection. **Day 6** ● Histological confirmation of cardiac myxoma. **Month 1** ● Follow-up Doppler echocardiography demonstrated adequate recovery, mild mitral regurgitation, and no tumor recurrence. ● Patient is asymptomatic.

Risk stratification scales were calculated, obtaining 189 and 4 points in the GRACE and TIMI scores, respectively. Initial management included double antiplatelet therapy with aspirin and clopidogrel, and anticoagulation with enoxaparin. The atrial fibrillation reverted with the administration of Lanatoside C.

## Diagnostic Assessment

**FIGURE 1:**
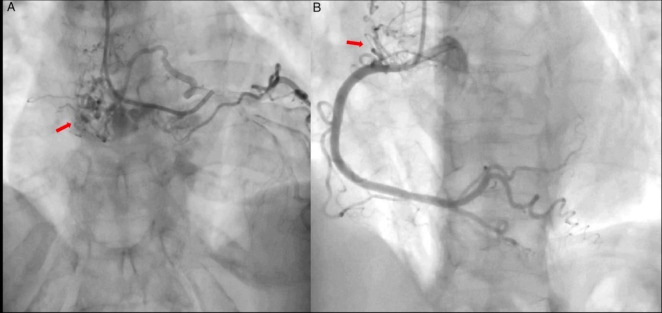
Coronary angiography. (A) Left coronary artery showing abnormal vascular structures (arrow) suggestive of arteriovenous fistula and a highly vascularized mass. Marginal branch demonstrates proximal 90% obstruction (Supplementary Video 1). (B) Right coronary artery shows no obstruction, abnormal vascular structures are also present (arrow).

The patient was initially managed as a NSTEMI, and an urgent coronary angiography (CA) was performed in order to discard coronary artery occlusion. The study showed abnormal and significant neovascularization within the left atrium and a 90% obstruction at the origin of the marginal branch, which suggested neovascularization of an atrial mass. (Figures 1A,B and Supplementary Video 1).

**FIGURE 2:**
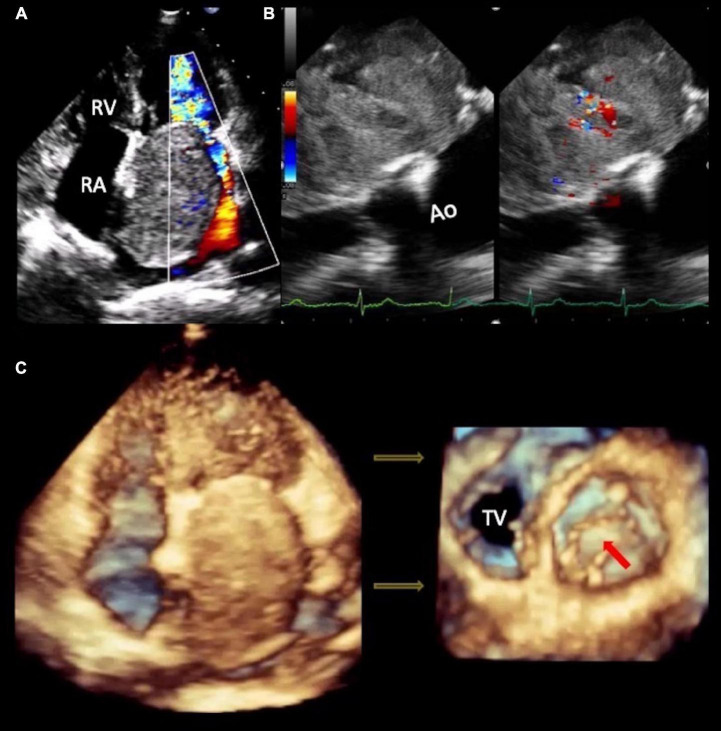
Echocardiogram. (A) 2D-TTE four-chamber view shows a large echogenic oval mass that occupies practically the entire left atrium, with regular borders, heterogeneous echogenicity, adhered to the interatrial septum by a short pedicle and moderate to severe mitral regurgitation in the color Doppler. (B) 2D-Transesophageal echocardiogram and color-Doppler evidenced significant vascularization inside the mass. (C) 3D-TTE shows the mass protruding through the mitral valve into the left ventricle during end-diastole (Supplementary Video 2). Ao, aorta; RA, right atrium; RV, right ventricle; TTE, transthoracic echocardiogram; TV, tricuspid valve.

Transthoracic echocardiogram (TTE) showed a large echogenic oval mass in the left atrium attached to the interatrial septum by a short pedicle measuring 5 × 7 × 6 cm, as well as moderate to severe mitral regurgitation (MR) by color-Doppler. The biventricular systolic function was normal (LVEF-63% and TAPSE-21 mm). Two dimensional-Doppler-transesophageal echocardiography showed a giant vascularized heterogeneous mass. 3D-TTE accurately identified the mass protruding through the mitral valve into the left ventricle during end-diastole and the 2D-TTE color flow and continuous wave Doppler in four chamber view showed a diastolic mitral peak velocity of 2.08 m/s, maximum gradient of 17 mmHg and mean gradient of 10.33 mmHg (Figures 2A–C and Supplementary Video 2).

**FIGURE 3:**
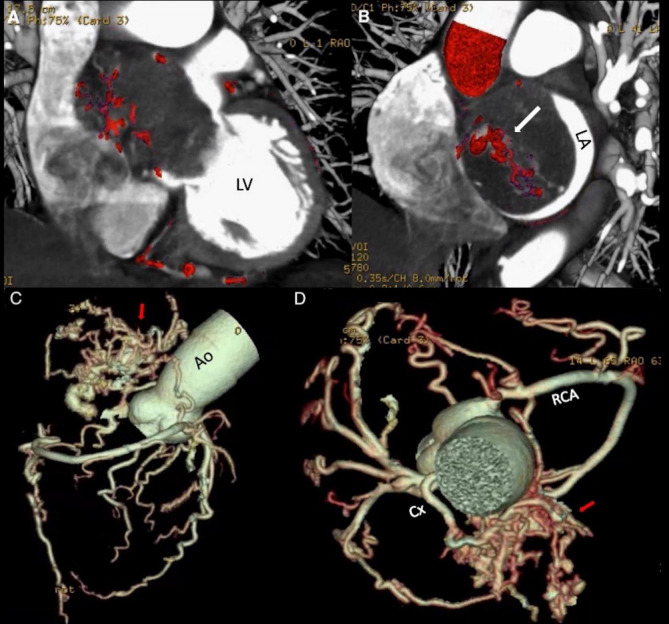
(A,B) Coronary Computed Tomography Angiography. Hypodense mass located in the left atrium attached to the interatrial septum, with a dense network of blood vessels within. The intra- atrial mass protrudes through the mitral valve into the left ventricle during diastole, causing impairment of the atrial emptying and mitral valve closure. (C,D) Coronary Computed Tomography Angiography reconstruction. Complex blood vessel network (arrows) anastomosed to branches of the circumflex artery and the right coronary artery (Supplementary Video 3). Ao, aorta; Cx, circumflex artery; LA, left atrium; LV, left ventricle; RCA, right coronary artery.

After the echocardiographic findings, the heart team agreed on performing a CCTA, which showed a non-infiltrative and very vascularized mass attached to the interatrial septum (Figures 3A,B). A 3D-CCTA reconstruction additionally showed the significant vascularity of the tumor, characterized the anatomy of the coronary arteries and the origin of the neovascularization (Figure 3C). Double arterial blood supply was observed, with anomalous vessels arising from the right coronary artery and the circumflex artery (Figure 3D and Supplementary Video 3).

**FIGURE 4:**
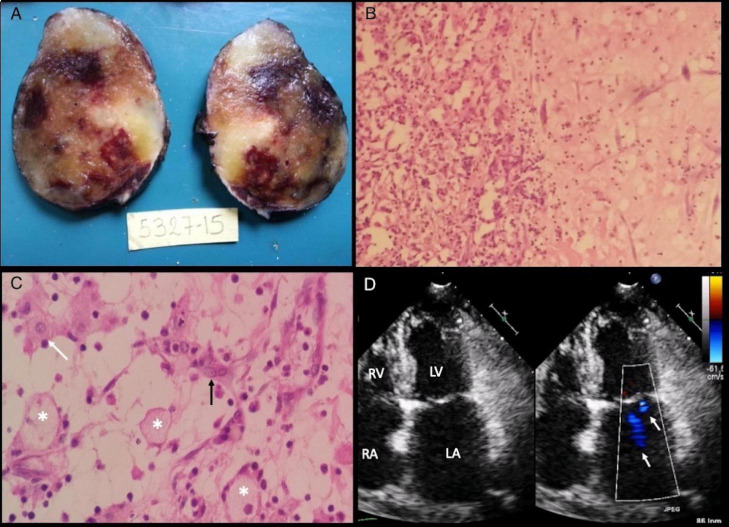
Macroscopic and microscopic specimen findings. (A) Oval mass of 80 grams, 7 × 4 × 5 cm of diameter with a smooth surface showing white/yellow colors with zones of hemorrhagic aspect. Intact capsule that delimits the entire mass, compatible with its benign and non-invasive nature. (B) On the left side, a pattern of glanduloid/vascular cavities with abundant inflammatory lymphocytic infiltrate. On the right side, homogeneous stroma with clear spaces and isolated myxoma cells with fusi morphology. H/E10x (C) Myxoma’s stroma shows decreased cellular density and multiple blood vessels (asterisks). Stellate appearance cells present eosinophilic cytoplasm and round nucleus (white arrow). Syncytial Cells with a string morphology (black arrow). H/E 40x. (D) Follow-up echocardiogram. 2D and color flow TTE in four-chamber view with 2 jets of mild mitral regurgitation (white arrows) and no cardiac mass in the left atrium. Abbreviations as before.

The patient was scheduled for surgical resection, which consisted of median sternotomy, and bicaval bypass with cardioplegic cardiac arrest. Before the tumor resection, the surgical team ligated both feeding arteries. An oval mass presenting multiple hemorrhagic areas within was removed and the final measurement was 7 × 4 × 5 cm. Pathologic findings were consistent with a LAM (Figures 4A–C).

One month after surgery, a 2D-TTE demonstrated a diastolic mitral peak velocity of 0.67 m/s, maximum gradient of 1.79 mmHg, and mean gradient of 1.07 m/s and mild MR (Figure 4D). Atrial fibrillation and tumor recurrence were not detected in the following 18 months of follow-up.

## Discussion

The clinical presentation of left atrial tumors depends on location and size, and whether it is associated with valvular insufficiency, obstruction, or embolic events. The initial assessment of atypical presentations may be challenging. Regarding the case, the mechanisms responsible for the origin of the EKG findings and manifestations could be due to two different situations. Fast AF and hypotension may have resulted in an oxygen supply demand imbalance (type 2 NSTEMI), thus the absence of findings in the CA that could explain the ST segment abnormalities and the complete recovery after surgical removal. Similarly, LAM should always be considered as an embolic source in healthy patients with systemic thromboembolism, including coronary artery embolization, which is extremely rare (0.06%) (6). Spontaneous recanalization prior to CA could also explain the reported findings.

In any case, the cardiac territory supplied by an obstructed artery is at increased risk of hypoxic cell injury, as seen during CA in the marginal branch, which showed a concomitant luminal reduction of 90%, which may have further contributed to the initial presentation in our patient.

Imaging evaluation of a cardiac mass includes echocardiography as the initial study to determine its location, size, and mobility. Other imaging modalities are required to visualize the characteristics of the coronary arteries and their relationship to the neovascularization that can be found in myxomas, however, these techniques may not be routinely available in some countries and were highly underused a few years ago, as described by Elbardissi et al. (7), when they evaluated 278 patients with cardiac masses, which were mainly diagnosed by echocardiography and only a few underwent CA (*n* = 33, 10%) or CCTA (*n* = 9, 3%). Case series have shown a relatively high rate of low neovascularization in myxomas, with an increased and standardized use of new imaging modalities such as cardiac magnetic resonance imaging (MRI) and CCTA for evaluating this type of tumors, either as a complementary tool of CA or by replacing it (3–5). CCTA overcomes the limitations of echocardiography and CA by displaying detailed images of the origin and morphology of tortuous and dilated blood vessels; and, at the same time, reliably ruling out coronary artery disease (CAD) in patients scheduled for surgical resection (4).

Kim et al. (5) described 2 patients who were initially diagnosed with LAM by echocardiography and subsequently underwent preoperative CCTA instead of coronary angiography to rule out concomitant CAD, which provided more information about the vasculature of the mass. On the other hand, this technique avoids the risk of embolization and other complications of CA and can provide information that modifies the chosen surgical method, such as ligation of the feeding arteries before surgical resection (4).

Differential diagnosis of LAM mainly includes cardiac malignant tumors such as angiosarcoma and thrombi. Large thrombi may represent a diagnostic challenge by using echocardiography alone, but characteristic findings include attachment to the posterior left atrial wall, a broad base and tend to be immobile, contrary to LAM. Additionally, malignant tumors tend to be highly vascularized masses which can be observed by using contrast echocardiography, which provides specific information for differentiating malignant from benign neoplasms. If low vascularization is present, slow contrast filling of the mass would be observed, as usually occurs in benign tumors, such as cardiac myxomas. In this particular case, due to the extreme vascularization, a contrast agent would show a rapid filling and late opacification, simulating a malignant process, which emphasizes the importance of pathologic findings for the final diagnosis of a cardiac tumor. Regarding thrombi, which are usually not vascularized, contrast echocardiography would show no filling of the contrast agent (8).

As mentioned, the pathological findings represent a fundamental tool for the final diagnosis of a cardiac mass, whereas a multi-imaging evaluation helps in the assessment of the morphology and characteristics of the tumor and its relationship with the surrounding cardiac and paracardiac structures, and therefore is particularly useful for the surgeon in order to precisely plan the surgical intervention.

In our case, CCTA showed that the tumor’s enormous blood supply came from the right coronary and circumflex arteries, which helped the heart team understand the complexity of the myxoma vasculature and ligate both feeding arteries before the mass resection. Therefore, CCTA may confer a better alternative than CA in the evaluation of cardiac masses and in their differential diagnosis, especially in the context of a highly vascularized myxoma and when the patient is older than 40 years to rule out concomitant CAD.

On the other hand, cardiac MRI provides images suitable for the same purpose without exposing the patient to ionizing radiation, but it relies on patient cooperation, implanted magnetic devices, and is less useful than CCTA in evaluating coronary arteries during an acute event, which may be very important in the context of LAM, specifically in urgent atypical presentations as the one described in this case.

For the preoperative evaluation of myxomas, an imaging modality such as MRI, CCTA or CA must be performed in order to better characterize the mass and evaluate CAD. Preoperative CCTA represents an accurate method to assess myxoma morphology, vascularization, and avoids CA risks. Three-dimensional reconstructions may be even more useful in the decision making of the surgical team.

## Data Availability Statement

The datasets for this article are not publicly available due to concerns regarding participant/patient anonymity. Requests to access the datasets should be directed to the corresponding author.

## Ethics Statement

The studies involving human participants were reviewed and approved by the Research and Ethics Committee of National Institute of Cardiology Ignacio Chavez. The patients/participants provided their written informed consent to participate in this study. The patients/participants provided their written informed consent for the publication of the case report, and for the publication of any potentially identifiable images or data included in this article.

## Author Contributions

JS-R, SS-A, and NE-Z collected the data and headed the elaboration of the present manuscript. VF-B, ES-P, and AE-G reviewed the recent literature. JA-F performed the echocardiogram and analyzed the CCTA. JB and MG-C carefully revised the manuscript. AA-F interpreted the macroscopic and microscopic images. All authors contributed to the conception, analysis, and design of the complete case report, revising of the manuscript, enhancing its intellectual content, and approving the final version of the manuscript.

## Conflict of Interest

The authors declare that the research was conducted in the absence of any commercial or financial relationships that could be construed as a potential conflict of interest.

## Publisher’s Note

All claims expressed in this article are solely those of the authors and do not necessarily represent those of their affiliated organizations, or those of the publisher, the editors and the reviewers. Any product that may be evaluated in this article, or claim that may be made by its manufacturer, is not guaranteed or endorsed by the publisher.
